# Ebola virus infection modeling and identifiability problems

**DOI:** 10.3389/fmicb.2015.00257

**Published:** 2015-04-09

**Authors:** Van Kinh Nguyen, Sebastian C. Binder, Alessandro Boianelli, Michael Meyer-Hermann, Esteban A. Hernandez-Vargas

**Affiliations:** ^1^Systems Medicine of Infectious Diseases, Department of Systems Immunology and Braunschweig Integrated Centre of Systems Biology, Helmholtz Centre for Infection ResearchBraunschweig, Germany; ^2^Department of Systems Immunology and Braunschweig Integrated Centre of Systems Biology, Helmholtz Centre for Infection ResearchBraunschweig, Germany; ^3^Institute for Biochemistry, Biotechnology and Bioinformatics, Technische Universität BraunschweigBraunschweig, Germany

**Keywords:** Ebola, mathematical modeling, kinetics, viral dynamics, identifiability, EBOV

## Abstract

The recent outbreaks of Ebola virus (EBOV) infections have underlined the impact of the virus as a major threat for human health. Due to the high biosafety classification of EBOV (level 4), basic research is very limited. Therefore, the development of new avenues of thinking to advance quantitative comprehension of the virus and its interaction with the host cells is urgently needed to tackle this lethal disease. Mathematical modeling of the EBOV dynamics can be instrumental to interpret Ebola infection kinetics on quantitative grounds. To the best of our knowledge, a mathematical modeling approach to unravel the interaction between EBOV and the host cells is still missing. In this paper, a mathematical model based on differential equations is used to represent the basic interactions between EBOV and wild-type Vero cells *in vitro*. Parameter sets that represent infectivity of pathogens are estimated for EBOV infection and compared with influenza virus infection kinetics. The average infecting time of wild-type Vero cells by EBOV is slower than in influenza infection. Simulation results suggest that the slow infecting time of EBOV could be compensated by its efficient replication. This study reveals several identifiability problems and what kind of experiments are necessary to advance the quantification of EBOV infection. A first mathematical approach of EBOV dynamics and the estimation of standard parameters in viral infections kinetics is the key contribution of this work, paving the way for future modeling works on EBOV infection.

## 1. Introduction

Ebola was characterized for the first time in 1976 close to the Ebola River located in the Democratic Republic of the Congo (WHO, 1978). Since then, outbreaks of EBOV among humans have appeared sporadically causing lethal diseases in several African countries, mainly in Gabon, South Sudan, Ivory Coast, Uganda, and South Africa (CDC, 2014). Among the most severe symptoms of the EBOV disease are fever, muscle pain, diarrhea, vomiting, abdominal pain and the unexplained hemorrhagic fever (Calain et al., 1999). Fatalities are predominantly associated with uncontrolled viremia and lack of an effective immune response. However, the pathogenesis of the disease is still poorly understood (Peters and Peters, 1999; Feldmann et al., 2003).

**Figure 1:**
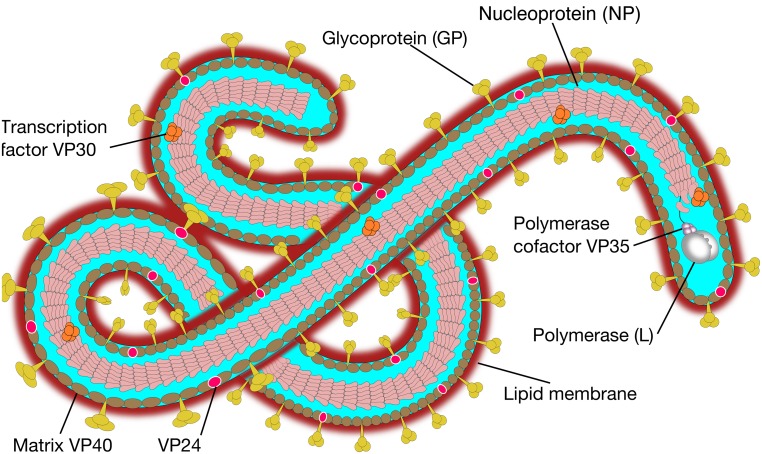
**Ebola virus molecular structure**. The Ebola genome is composed of 3 leader, nucleoprotein (NP), virion protein 35 (VP35), VP40, glycoprotein (GP), VP30, VP24, polymerase (L) protein and 5 trailer (adapted from SIB SWISS Institute of Bioinformatics, 2014).

Ebola virus belongs to the family of *Filoviridae*, from Latin *filum* which means thread (Carter and Saunders, 2013). Ebola virus is classified in Tai Forest, Sudan, Zaire, Reston, and Bundibugyo. The human Ebola epidemics have been mainly related to infection by the Zaire and Sudan strains. Filovirus virions possess several shapes, a property called pleomorphism (Feldmann et al., 2003). These shapes are appearing as either U-shaped, 6-shaped, or other configurations, e.g., Figure 1.

The natural hosts of EBOV still remain unsettled, but it is tenable that EBOV persists in animals which transmit the virus to non-human primates and humans (Knipe et al., 2001). It has been reported that fruit bats are capable of supporting EBOV replication without becoming ill and may serve as a major reservoir (Swanepoel et al., 1996; Knipe et al., 2001; Leroy et al., 2009; Formenty, 2014). EBOV can spread from an infected person to others through direct contact with blood or body fluids (e.g., saliva, sweat, feces, breast milk, and semen), objects (i.e., needles) that have been contaminated with the virus and infected fruit bats or primates (Peters and Peters, 1999; Feldmann et al., 2003; CDC, 2014). The 2014 Ebola epidemic is the largest ever reported in history, affecting multiple countries in West Africa and being imported to other countries: one infection case was reported in Spain while in the United States one death and two locally acquired cases in healthcare were reported (CDC, 2014).

EBOV can infect a wide variety of cell types including monocytes, macrophages, dendritic cells, endothelial cells, fibroblasts, hepatocytes, adrenal cortical cells, and several types of epithelial cells, all supporting EBOV replication. Monocytes, macrophages, and dendritic cells are early and preferred replication sites of the virus (Knipe et al., 2001). Furthermore, murine studies have revealed that EBOV can infect cells in different compartments, showing high viral titers in liver, spleen, kidney and serum (Mahanty et al., 2003).

Due to its high infectivity and fatality, the virus is classified as a biosafety level-4 agent, restricting basic research for Ebola disease (Halfmann et al., 2008). Infection parameters and quantification of the interactions between the virus and its target cells remain largely unknown. Therefore, the development of new avenues of thinking to bring forward quantitative comprehension of the relationship between the virus and the host is urgently needed. To this end, mathematical models can help to interpret experimental results on quantitative grounds. Model simulations can infer predictions to initiate further and conclusive experiments that may solve relevant scientific questions and advance knowledge of EBOV infection.

Recently, mathematical models have played a central role to capture the dynamics of different virus infections (Nowak and May, 2000). Among the most popular are HIV (Kirschner, 1996; Wu et al., 1998; Duffin and Tullis, 2002; Perelson, 2002; Hernandez-Vargas et al., 2010; Hernandez-Vargas and Middleton, 2013; Jaafoura et al., 2014), hepatitis virus (Ribeiro et al., 2002; Reluga et al., 2009; Guedj et al., 2013) and influenza virus infection models (Baccam et al., 2006; Handel et al., 2010; Smith and Perelson, 2011; Pawelek et al., 2012; Hernandez-Vargas et al., 2014). These models have been instrumental to study the mechanisms that control viral kinetics in order to provide a quantitative understanding and to formulate recommendations for treatments. Similarities of parameter values for EBOV infection to other viral infections that promote outbreaks, e.g., influenza virus infection, could be expected. Nevertheless, to the best of our knowledge, there has not been any mathematical approach until now to describe EBOV dynamics. This and the interaction of EBOV virus with non-human primate epithelial cells is the key contribution of this work.

## 2. Materials and methods

### 2.1. Mathematical model

**Figure 2:**
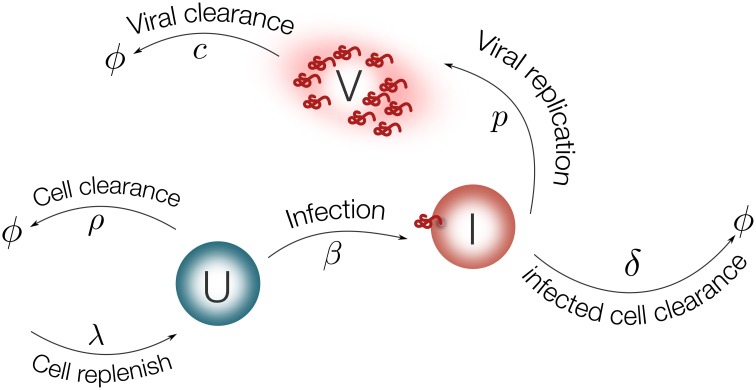
**Schematic representation of the model for EBOV infection**. Target cells (*U*) are replenished with rate λ and die with rate ρ. Virus (*V*) infects target cells (*U*) with rate β. Infected cells are cleared with rate δ. Once cells are productively infected (*I*), they release virus at rate *p* and virus particles are cleared with rate *c*.

The mathematical model proposed here to represent EBOV dynamics is based on the well established target cell-limited model (Nowak and May, 2000), see Figure 2. This has served to model several viral diseases, among them HIV infection (Wu et al., 1998; Perelson, 2002), hepatitis virus infection (Ribeiro et al., 2002) and influenza virus infection (Baccam et al., 2006; Hernandez-Vargas et al., 2014). A detailed reference for modeling of viral dynamics can be found in Nowak and May (2000).

Using ordinary differential equations (ODEs), the EBOV infection model is considered as follows:

(1) $$\frac{dU}{dt}=\lambda -\rho U-\beta UV$$

(2) $$\frac{dI}{dt}=\beta UV-\delta I$$

(3) $$\frac{dV}{dt}=pI-cV$$

EBOV target cells can be either in a susceptible (*U*) or an infected state (*I*). Cells are replenished with a constant rate λ and die with rate ρ. Note that the condition λ = *U*_0_ρ should be satisfied to guarantee homeostasis in the absence of viral infection, such that only ρ is a parameter to be determined. Virus (*V*) infects susceptible cells with rate constant β. Infected cells are cleared with rate δ. Once cells are productively infected, they release virus at rate *p* and virus particles are cleared with rate *c*.

**Figure 3:**
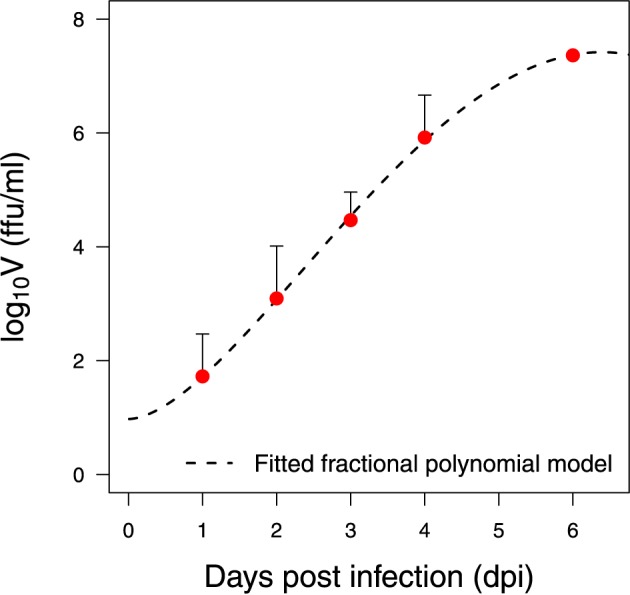
**Data preparation**. Fitted statistical model for the wild-type Vero cells infected with EBOV at a *low multiplicity of infection* (MOI) (Halfmann et al., 2008)

The initial number of susceptible cells (*U*_0_) can be taken from the experiment in Halfmann et al. (2008) as 5 × 10^5^. The initial value for infected cells (*I*_0_) is set to zero. The viral titer in Halfmann et al. (2008) is measured in foci forming units per milliliter (*ffu/ml*). The initial viral load (*V*_0_) is estimated from the data using the fractional polynomial model of second order (Royston and Altman, 1994). The best model based on the Akaike Information Criterion (AIC) is presented in Figure 3, providing an estimate of 9 *ffu/ml* for *V*_0_. The parameter ρ is fixed from literature as 0.001 day^−1^ (Moehler et al., 2005). The effect of fixing this value on the model output is evaluated with a sensitivity analysis.

### 2.2. Experimental data

As described in the previous section, this paper is mainly focused on the interaction between the virus and the target cells. A safe way to study the virus life cycle was proposed in Halfmann et al. (2008). The disease pathogenesis of EBOV in non-human primates is known to be more faithful in portraying the human condition than in rodents (Knipe et al., 2001). Replication kinetics of EBOV are studied in Vero cells, a cell line derived from kidney epithelial cells of African green monkeys (Halfmann et al., 2008). This non-human primate is a known source of *Filoviridae* virus infection, e.g., the European Marburg outbreak from 1967 (Knipe et al., 2001). Wild-type Vero cells and a Vero cell line expressing VP30 were tested to reveal their ability to confine EBOV to its complete replication cycle. In this study, viral kinetics for wild-type Vero cells infected with EBOV at different multiplicities of infection (MOI) were considered (Halfmann et al., 2008). The viral growth data is presented in Figure 3. Further details on the data, methods and experiments can be found in Halfmann et al. (2008).

### 2.3. Parameter estimation

Parameter fitting is performed minimizing the root mean square (RMS) difference on log scale between the model output, ŷ_*i*_, and the experimental measurement, *y*_*i*_:

(4) $$\text{RMS}=\sqrt{\frac{1}{n}\sum_{i\;=\;1}^{n}{\left(\log_{10}y_{i}-\log_{10}{\hat{y}}_{i}\right)}^{2}}$$

where *n* = 5 (Halfmann et al., 2008) is the number of measurements. Differential equations are solved by R 3.1.2 (R Core Team, 2014) using the deSolve package (Soetaert et al., 2010). The minimization of RMS is performed using the Differential Evolution (DE) algorithm employing the DEoptim package (Storn, 1997; Mullen et al., 2011). The DE global optimization algorithm does not rely on initial parameter guesses and converged faster than the other tested methods, including genetic algorithms and the quasi-Newton (BFGS, L-BFGS-B) algorithms.

### 2.4. Parameter uncertainty

Viral load variability is very large for several viral infectious diseases (Mahanty et al., 2003; Baccam et al., 2006; Toapanta and Ross, 2009; Groseth et al., 2012). In order to consider the large variability of biological problems, a bootstrap method is applied to the data series presented in Halfmann et al. (2008). Bootstrapping is a statistic method for assigning measures of accuracy to estimates (Davison and Hinkley, 1997; Xue et al., 2010). The nonparametric bootstrap requires data to be independent and identically distributed while the parametric bootstrap requires to impose on the data a distribution assumption which is usually unknown. For the data in Halfmann et al. (2008), three bootstrap approaches were considered: (i) the conventional parametric approach assumes a log-normal distribution of the measurement, (ii) the nonparametric approach assumes uniform distribution in the measurement range, and (iii) the weighted bootstrap assigns to the cost function a vector of random weights from exponential distribution with mean one and variance one (Ma and Kosorok, 2005; Xue et al., 2010).

For each repetition, the model parameters are refitted to obtain the corresponding parameter distribution. The 95% confidence interval of parameter estimates is computed using the outcome of the bootstrap method (Xue et al., 2010). For each parameter, the 2.5 and 97.5% quantiles of the estimates are used to form the 95% confidence interval.

### 2.5. Parameter identifiability and sensitivity

A critical obstacle to overcome in mathematical modeling is how to verify whether model parameters are identifiable based on the measurements of output variables (Xia, 2003; Xia and Moog, 2003; Wu et al., 2008; Miao et al., 2011). A system that is algebraically identifiable may still be practically non-identifiable if the amount and quality of the measurements is insufficient and the data shows large deviations. The novel approach proposed in Raue et al. (2009) exploits the profile likelihood to determine identifiability and is considered here. This method is able to detect both structurally and practically non-identifiable parameters.

Identifiability properties are studied for the model Equations (1–3) and the data set in Halfmann et al. (2008). The idea behind this approach is to explore the parameter space for each parameter θ_*i*_ by re-optimizing the RMS with respect to all other parameters θ_*j* ≠ *i*_. In particular, for each parameter θ_*i*_, a wide range of values centered at the optimized value is generated in an adaptive manner. Re-optimization of RMS with respect to the other parameters is done for each value of parameter θ_*i*_. The main task is to detect directions where the likelihood flattens out (Raue et al., 2009). The resulting profiles are plotted vs. each parameter range to assess the parameter identifiability visually.

In model fitting, some parameters may have little effect on the model outcome, while other parameters are so closely related that simultaneous fitting could be a difficult task. For this aspect, the scatter plots using pairs of parameters over different bootstrap replicates will be reported. Furthermore, sensitivity analysis of the estimated parameters is performed (Brun et al., 2001; Soetaert, 2014). For each data point the derivative of the corresponding modeled variable value with respect to the selected parameter is computed. The normalized sensitivity function reads as

(5) $$\frac{\partial y_{i}}{\partial \Theta_{j}}\cdot \frac{w_{\Theta_{j}}}{w_{y_{i}}}$$

where *y*_*i*_ denotes the model variables, Θ_*j*_ is the parameter of interest, and the ratio *w*_Θ_*j*__/*w*_*y*_*i*__ is the normalized factor corresponding to its nominal value (Soetaert and Petzoldt, 2010). Summary statistics of the sensitivity functions can be used to qualify the impact of the parameter on the output variables, i.e., the higher the absolute value of the sensitivity summary statistics, the more important the parameter (Brun et al., 2001). For the model in Equations (1–3), the sensitivity functions will be plotted vs. time to illustrate the parameters' role on the model output. The parameters that have little effect do not need to be fine-tuned extensively in model fitting.

### 2.6. Cross-validation

It is important to prove how the model predictions will generalize to an independent data set, revealing how accurately the predictive value of a model is in practice. In this paper, the parameter set obtained from the data of wild-type Vero cells infected at low MOI is used to predict the replication kinetics of the data at high MOI presented in Halfmann et al. (2008).

## 3. Results

Although significant progress has been made to the identification and characterization of EBOV, human data is very limited due to the long asymptomatic periods of the virus and its high mortality. Animal models are pivotal to shed light on this lethal disease. Due to the very close similarities with the human immune system, non-human primates are the preferred animal model for several viral infections e.g., HIV). Moreover, EBOV infection has been adapted to guinea pigs and mice (Feldmann et al., 2003), serving as a flexible model in comparison to human and non-human primates. In this work, we focus on the interaction between the virus and the host cells. *In vitro* data can be very convenient due to the important simplification of the *in vivo* complexity of biological problems. Thus, for parameter fitting procedures, we consider the experimental data from Halfmann et al. (2008), which investigates EBOV kinetics in a Vero cell line.

Before rigorous optimization methods can be applied to estimate the model parameters using experimental data, the verification of parameter identifiability is required. The omission of identifiability analyses may result in incorrect fits and consequently incorrect interpretations. The identifiability analysis in the model Equations (1–3) has been broadly studied (Xia, 2003; Xia and Moog, 2003; Wu et al., 2008; Miao et al., 2011; Hernandez-Vargas et al., 2014). All parameters in the model Equations (1–3) were shown to be algebraically identifiable given measurements of viral load and initial conditions (*U*_0_, *I*_0_, and *V*_0_) (Wu et al., 2008). However, the difference between structural identifiability and practical identifiability in the presence of measurement error requires further identifiability studies. To address practical identifiability, the approach proposed by Raue et al. (2009) is considered here for the data presented in Figure 3.

**Figure 4:**
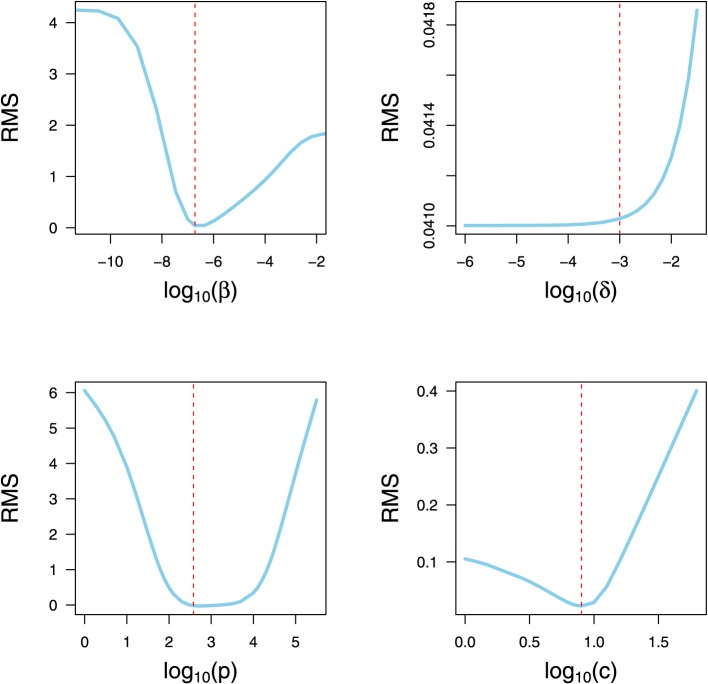
**Parameter Identifiability**. RMS profile of model parameters. Each parameter is varied in a wide range around the optimized value. Subsequently, the DE algorithm is used to refit the remaining parameters to the data set of Halfmann et al. (2008). The vertical dashed lines indicate the value obtained from the optimization for all four parameters collectively.

The resulting RMS profiles in Figure 4 for β, *p* and *c* show a convex shape of which the optimization routine can reach their minimum. Note that the profile of δ is flat in one tail, suggesting that parameter δ can be chosen arbitrarily small without affecting the fit quality (Raue et al., 2009). In spite of this, the lower bound of this parameter has a clear biological constraint. To be precise, the half-life of an infected cell cannot be longer than that of an uninfected cell. There is experimental evidence that the half-life of epithelium cells in lung is 17–18 months in average (Rawlins and Hogan, 2008). In view of this, the infected cell death rate (δ) is fixed at 10^−3^.

**Figure 5:**
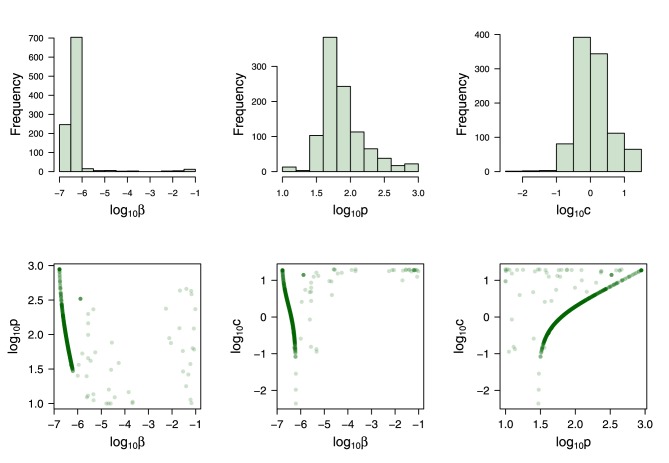
**Weighted bootstrap results. Top row**: Distributions from 1000 sample estimates are presented for the three parameters: β, *p* and *c*. **Bottom row**: Scatter plot between bootstrap parameters. The parameter ρ is fixed during the bootstrapping at 0.001 (Moehler et al., 2005). Numerical values for the model Equations (1–3) are presented in the Table 1.

Bootstrapping can provide more insights into the distribution of parameter values based on experimental data in Halfmann et al. (2008). For the sake of clarity, we present only the weighted bootstrap (Xue et al., 2010) in the results, the other two methods can be found in the supplementary material. Distributions of the model parameters are shown in Figure 5. Bootstrap estimates for the viral clearance (median *c* = 1.05 *day*^−1^) is slightly below other viral infection results (Table 1). For example, clearance of influenza virus varied from 2.6 to 15 *day*^−1^ in (Baccam et al., 2006; Miao et al., 2011; Pawelek et al., 2012; Hernandez-Vargas et al., 2014). This may be attributed to the fact that the viral clearance is computed for *in vitro* experiments.

**Table 1:** Estimates of infection parameters^*^.

**Parameters (units)**	Best fit^**^	**Bootstrap estimates**		
		**2.5% quantile**	**Median**	**97.5% quantile**
β [day^−1^ (ffu/ml)^−1^10^−7^]	1.91	1.78	4.06	261.95
*p* (ffu/ml day^−1^ cell^−1^)	378	31.80	62.91	580.69
*c* (day^−1^)	8.02	0.18	1.05	18.76
*R*_0_ × 10^3^	4.52	4.19	13.01	116.06
*t*_inf_ (hours)	5.64	1.68	9.49	10.79

* *Note that these parameter should be interpreted with the discussed identifiability problems*.

** *Values obtained from optimization procedure to the low MOI viral titer presented in Halfmann et al. (2008)*.

EBOV is known to replicate at an unusually high rate that overwhelms the protein synthesis of infected cells (Sanchez, 2001). Consistent with this observation, bootstrap estimates revealed a very high rate of viral replication, *p* = 62 (95% CI: 31−580) (Table 1). Although the scatter plot in Figure 5 shows that the estimate of *p* can be decreased given a higher effective infection rate (β), a replication rate of at least 31.8 ffu/ml cell^−1^ day^−1^ is still needed to achieve a good fit of the viral replication kinetics in Figure 3.

Scatter plots are a graphical sensitivity analysis method, and a simple but useful tool to test the robustness of the results. The estimated parameters are plotted against each other. Scatter plots for the parameters in Figure 5 provide visual evidence that these parameters strongly depend on one another such that their individual values can not be independently determined. That is, increasing the values of *p* increases the estimations of *c*. Decreasing the estimations of β increases the estimation of both *c* and *p*. However, the green curves in Figure 5 provide the most likely region where the parameters values can be found. In order to verify this intuition, we fix the viral clearance rate (*c*) at 4.2 (Miao et al., 2010) and then estimate the others two parameters (β and *p*). The results of 1000 bootstrap replicates reveal that fixing the parameter *c* improves the fitting with a narrow confidence interval (see Supplementary Materials 1.3).

**Figure 6:**
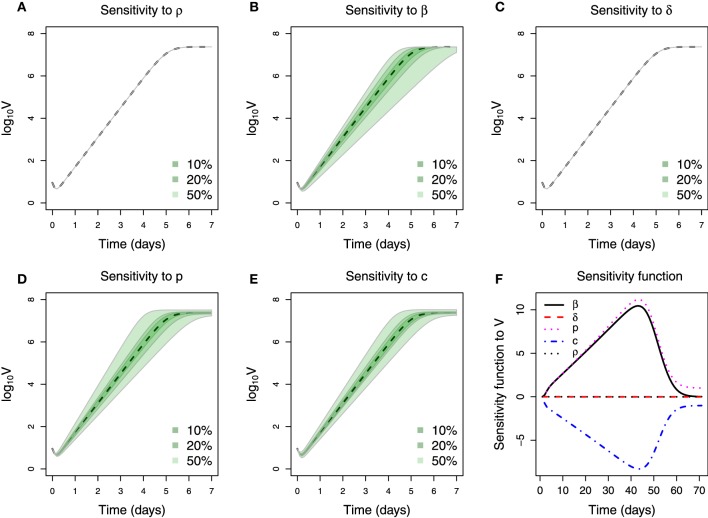
**Sensitivity of parameters. (A–E)** Plotting of viral titer variation vs. time. The dashed line is the viral kinetics obtain from nominal parameter values. Three color shades in each figure represent the viral load variation range when varying the corresponding parameter by a percentage denoted in the legend. **(F)** Parameters sensitivity function over time, the values in y-axis are calculated using Equation (5).

The sensitivity study for the mathematical model Equations (1–3) is performed in a similar fashion to Brun et al. (2001); Soetaert (2014). Figures 6A–E show the effect on the viral load when varying the respective parameter by 10, 20 and 50% around its nominal value. It can be seen that the healthy cell death rate (ρ), which in the virus-free steady state represents the cell turnover, has little effect on the viral load kinetics. This can be attributed to the fact that the experiment was performed *in vitro* and within a short period. Similarly, the effect of the infected cell death rate (δ) can also be neglected. This could be explained by the fact that the observed Ebola viral load was not decreasing (Figure 3), contrary to observations in other viral infections, e.g., influenza virus (Baccam et al., 2006). The remaining three parameters (β, *p*, and *c*) are sensitive, in the sense that a small change in parameter value can lead to a large difference in viral kinetics. Figure 6F summarizes in detail the parameter sensitivity functions. It is clear that the three parameters β, *p*, and *c* govern the infection kinetics while the effect of the two parameters ρ and δ can be neglected for this data set. Therefore, fixing both ρ and δ is adequate for the presented problem.

Moreover, both β and *p* can be seen as consistently increasing the viral load because their respective sensitivity functions are always positive, in contrast to the parameter *c*. Note that the absolute magnitude of change in the sensitivity functions of these three parameters is approximately equal over time (Figure 6F). The strong similarity in the sensitivity functions indicates that the corresponding parameters have equivalent effect on the viral titer. For instance, the sensitivity functions of β and *p* are very similar so that almost the same output of viral titer will be generated by increasing β if *p* is decreased correspondingly. A similar statement can also be made about the relationship between *c* and β.

**Figure 7:**
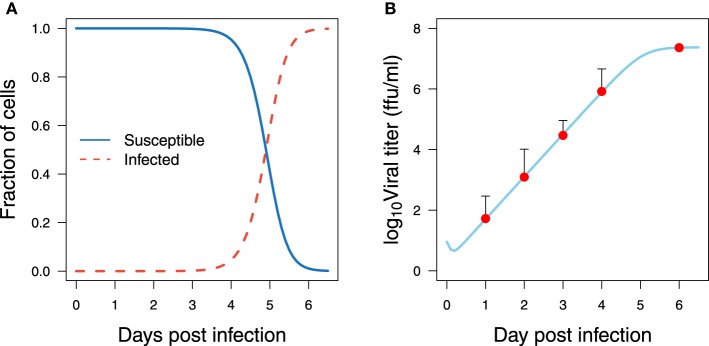
**Model fitting for EBOV kinetics**. Viral titer data with low MOI from Halfmann et al. (2008) and simulations from the best fit shown in Table 1 are in panel **(A)** for the host cells and **(B)** for the viral titer.

Computational simulations for the best fitting of the proposed mathematical model Equations (1–3) plotted in Figure 7B show that the virus grows exponentially from day 1 to 5 post infection. This is consistent with the mathematical analysis developed in Nowak et al. (1996), which deduced that the virus initially grows exponentially and can be better modeled as exp(*r*_0_*t*) while the susceptible cell population remains relatively constant, where *r*_0_ is the leading eigenvalue which solves the equation *r*^2^_0_ + (*c* + δ)*r*_0_ − (β*pU*_0_ − *c*δ) = 0.

Viral titer peaks at high levels, more than 10^7^ *ffu/ml*, which in general is 10 fold higher than those reported in influenza virus infection (Toapanta and Ross, 2009; Hernandez-Vargas et al., 2014). In addition, the viral titer reaches a plateau at day 6 and may remain at those levels (Figure 7B). No depletion of infected cells is observed in the period of observation. This could be a combined effect attributed to either high infection rate or high replication rate, and to the slow clearance of infected cells. To achieve virus titer levels as reported in Halfmann et al. (2008), either a high infection rate (β) of susceptible cells, or a high replication rate is required (Figure 5). Note that even though these estimations were performed *in vitro*, *in vivo* murine studies for EBOV infection (Mahanty et al., 2003) showed similar kinetics and time scales as those presented in Figure 7B.

### 3.1. Transmission measures

Infectivity is a critical parameter to assess the ability of a pathogen to establish an infection (Diekmann et al., 1990). To determine infectivity, we compute the reproductive number (*R*_0_), which is defined as the expected number of secondary infections produced by an infected cell in its lifetime (Diekmann et al., 1990; Heffernan et al., 2005). On the one hand, if *R*_0_ is less than one, each infected individual produces on average less than one infected individual, and therefore the infection will be cleared from the population. On the other hand, if *R*_0_ is greater than one, the pathogen is able to invade the susceptible population. This epidemiological concept can be applied to the model Equations (1–3) and computed as follows (Nowak et al., 1996):

(6) $$R_{0}=\frac{\lambda p\beta }{c\rho \delta }$$

**Figure 8:**
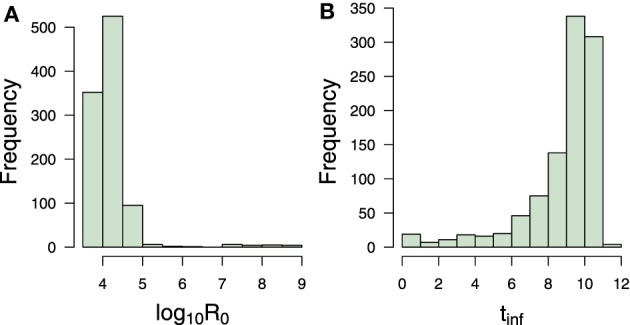
**Transmission measures**. Bootstrap estimate of **(A)** reproductive number and **(B)** *infecting time* in hours. Numerical values can be found in Table 1.

As expected, the estimated reproductive number in EBOV infection is very high, see Figure 8A and numerical results in Table 1. These results can be attributed to the fact that no depletion of virus was observed and to a slow clearance of infected cells. Thus, both parameters δ and *c* increase the value of *R*_0_. Note that very high estimates of the reproductive number in highly viremic influenza virus strains from *in vitro* experiments have also been reported, with an average of 1.7 × 10^3^ (Pinilla et al., 2012). It is worth to mention that fitting the model to *in vitro* data in Halfmann et al. (2008) could lead to small estimates for *c* and δ in comparison to an *in vivo* situation. Nevertheless, estimates of the epithelial cell half-life were 6 months in the trachea and 17 months in the lungs in average (Bowden, 1983; Rawlins and Hogan, 2008), which corresponds to a δ equal to 0.003 and 0.001, respectively. As mentioned previously, the δ was fixed at 0.001 in the computation of *R*_0_. Therefore, the estimated values of *R*_0_ interval are very likely to be positioned in a biologically plausible range, especially the upper bound. Notwithstanding, the estimate of *R*_0_ presented here should be interpreted with care within the limits of the data used.

Recent viral modeling works (Holder et al., 2011; Pinilla et al., 2012) have also introduced the term *infecting time*, which represents the amount of time required for a single infectious cell to cause the infection of one more cell within a completely susceptible population. Strains with a shorter infecting time have a higher infectivity (Holder et al., 2011; Pinilla et al., 2012). From model Equations (1–3), this measure can be computed as follows:

(7) $$t_{\text{inf}}=\sqrt{\frac{2}{p\beta U_{0}}}$$

Bootstrap results showed that EBOV possesses an average infecting times of 9.49 h (Table 1) which is approximately 7 times slower than the infecting time of influenza virus (Holder et al., 2011). This number provides a reasonable explanation for the kinetics of susceptible cells which slowly decrease from day 1 to day 4 (Figure 7A), and quickly deplete within the last 2 days. This number could also explain the absence of viral replication within the first 5.6 h after infection. This period corresponds to the short decreasing period observed in Figure 7B. The initial decrease of viral load thus can be attributed to self-clearance of the virus when some viruses have infected cells but are not yet able to replicate.

The infectivity parameters in Figure 8 characterize the EBOV infection kinetics in the data in Halfmann et al. (2008). The slow infection time of EBOV is compensated by its efficient replication. As a result, a short delay is followed by a massive amount of virus. The above infectivity parameters contributed an explanation for the high levels of viral load even when the susceptible cells were already depleted at the end of the experiment.

**Figure 9:**
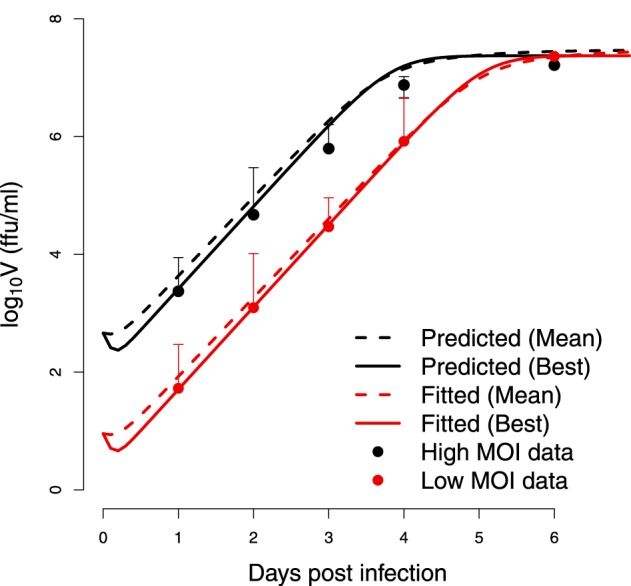
**Cross-validation**. Test of estimated parameters on an independent set of data. The viral replication kinetics in wild-type Vero cells infected with EBOV at a high multiplicities of infection (MOI) in Halfmann et al. (2008) are modeled starting from a higher initial viral load of *V*_0_ = 460 ffu/ml. The *(Mean)* indicates the predicted kinetics using parameters obtained from bootstrap while *(Best)* refers to the predicted kinetics using the parameters resulting from the optimization.

The best set of estimated parameters is challenged to validate the data at high multiplicities of infection (MOI) in Halfmann et al. (2008). The initial viral load is estimated using the fractional polynomial model of second order providing *V*_0_ at 460 ffu/ml. Figure 9 shows that the parameters derived from data at low multiplicity of infection are still consistent with data generated at high multiplicity of infection. The predicted kinetics follows the experimental data closely when changing the initial condition of the viral titer to 50 folds higher.

## 4. Discussion

Ebola virus (EBOV) is highly pathogenic for humans, being nowadays one of the most lethal pathogens worldwide. Ebola fatalities are predominantly associated with uncontrolled viremia and lack of an effective immune response (i.e., low levels of antibodies and no cellular infiltrates at sites of infection) (Feldmann et al., 2003).

The work presented here focused on the interaction between EBOV and the host cells, i.e., epithelial cells of green monkey. Experimental data on the Vero cell line from non-human primates could help to better understand the virus infection dynamics in humans (Knipe et al., 2001). However, the *in vitro* studies must be translated carefully to avoid over-interpretation to the *in vivo* context, which can sometimes lead to erroneous conclusions. Especially, the EBOV infection has been known to have abnormal behavior *in vivo* where different cells types and the immune system are involved (Knipe et al., 2001). Additionally, given the fact that EBOV exhibits an asymptomatic period in humans (Leroy et al., 2000), the viral dynamics model *in vivo* should take the eclipse phase into consideration. This feature can be modeled by adding an appropriate eclipse phase term as has been done previously (Moehler et al., 2005; Baccam et al., 2006). Nevertheless, given the problem of parameter identifiability exposed in the results, a complex model would not bring any better understanding. Once more data would become available, future work could attempt to address this issue, especially in the *in vivo* context.

The exposed identifiability issues in the results reveal the problematic of parameter estimation using solemnly the viral load measurements. Here, our efforts to cope thoroughly with the identifiability issues spotted the current restrictions on the estimated parameters. These restrictions cannot be resolved without the progress of new experiments, more measurements are necessary to sort out the identifiability problems presented here, e.g., measurements of infected and non-infected cells. Another possible experiment is to determine the EBOV clearance rate in the absence of target cells. For instance, Pinilla et al. (2012) employed an experiment in a similar fashion to determine the viral infectivity loss (*c*). Known influenza virus titers were incubated without target cells and followed up to determine the remaining infectious titers (Pinilla et al., 2012). In this way the approximate values of the viral clearance rate could be determined and provide a more accurate estimates for the whole set of kinetics parameters, as shown in the Supplemental Material 1.3.

The high EBOV replication reported here is in agreement with recent findings by Misasi and Sullivan (2014) as well as documented in Knipe et al. (2001), reporting that early and coordinated disruptions by Ebola genes and proteins (VP24, VP30, and VP35) lead to elevated levels of virus replication. The bootstrap results suggested that the EBOV average infecting time is approximately 9.5 h, at least 5 fold slower than estimations from influenza virus infection (Pinilla et al., 2012). These simulations outline the EBOV kinetics in the data from Halfmann et al. (2008), suggesting that a slow infecting time of EBOV is compensated by its efficient replication.

The model results suggested that the saturation of viral growth as observed in the data is induced by the loss of susceptible cells. This result has to be re-evaluated with a more complete data set, as the present data set would also be appropriately described by a logistic-growth model (data not shown) with an unspecific limitation of resources. However, a logistic model can explain only the growth behavior of the virus. As pointed out before (Wu et al., 2008), a higher resolution of the data and later time points which exhibit the long-term behavior of the viral load are required for a full determination of the mechanisms at work.

EBOV infection from *in vitro* and even murine systems may differ considerably from humans. The latency phase in human is much longer than in animals and EBOV symptoms in humans may appear from 2 to 21 days after exposure to the virus, having an average time of 8–10 days (Peters and Peters, 1999). Remarkably, mice infected by intra-peritoneal injection develop symptomatic infection where EBOV will increase rapidly at day 4 and continue to increase until day 6, with death occurring at day 6–7 post-infection (Mahanty et al., 2003). These experimental observations are compatible with our simulation results, suggesting that the growth of infected cells starts at day 3 post infection (Figure 7) while almost the whole susceptible cell pool is depleted at day 6 post infection. It is worth to mention that EBOV kinetics were similar in different tissue compartments (Mahanty et al., 2003): liver, spleen, kidney and serum. Consequently, further modeling approaches should address the EBOV kinetics in different compartments of the infected host.

The *in vitro* system may mimick a human context where the immune response against EBOV is not working adequately. The onset of a CD8+ T cell response as well as of the antibody response (Gupta et al., 2003) rely on early regulation of cytokines in the asymptomatic phase of the disease (Mahanty et al., 2003; Ebihara et al., 2006; García-Sastre and Biron, 2006). Human EBOV infection revealed that patients infected by the Sudan strain had lower levels of tumor necrosis factor TNF-α and interferon IFN-γ compared to those found in patients with fatal Zaire strain infection (Hutchinson and Rollin, 2007). Additionally, the levels of IFN-α were found significantly higher in surviving patients with Sudan strain infection (Hutchinson and Rollin, 2007), whereas the levels of IL-6, IL-8, IL-10, and macrophage inflammatory proteins were higher in patients with fatal infections (Hutchinson and Rollin, 2007). Therefore, modeling the effects of IFN-I would limit the number of infected cells by the introduction of a resistant state with a possible impact on the value of the viral replication rate (*p*). Future modeling studies need to quantify the situation *in vivo* where the effect of the immune system is taken into account.

The modeling work developed in this paper paves the way for future mathematical models and experiments to shed light on the reasons for less efficient control of Ebola virus infections. Determining empirically the EBOV clearance rate in the absence of target cells would fulfill the picture of EBOV kinetics *in vitro*. In addition, due to the critical relevance of the cytokine effects in EBOV pathogenesis, future modeling attempts should be directed to establish a more detailed model of interactions between the relevant cytokines and EBOV. Further insights into immunology and pathogenesis of EBOV will help to improve the outcome of this lethal disease.

### Conflict of interest statement

The authors declare that the research was conducted in the absence of any commercial or financial relationships that could be construed as a potential conflict of interest.
