# Supplementary material for: Ebola virus infection modeling and identifiability problems
Source: Front Microbiol. 2015 Apr 9;6:257. doi: 10.3389/fmicb.2015.00257 (PMC4391033; doi:10.3389/fmicb.2015.00257)
Supplement: Supplementary file 1 [file DataSheet1.PDF]

frontiers in Microbiology

Supplementary Material:

Ebola Virus Infection Modelling and Identifiability Problems

Van Kinh Nguyen [1], Sebastian C. Binder [2], Alessandro Boianelli [1], Michael Meyer-Hermann [2,3,*], Esteban A. Hernandez-Vargas [1,*]

[1] *Systems Medicine of Infectious Diseases, Department of Systems Immunology and Braunschweig Integrated Centre of Systems Biology, Helmholtz Centre for Infection Research, Braunschweig, Germany*

[2] *Department of Systems Immunology and Braunschweig Integrated Centre of Systems Biology, Helmholtz Centre for Infection Research, Inhoffenstr.7, 38124 Braunschweig, Germany*

[3] *Institute for Biochemistry, Biotechnology and Bioinformatics, Technische Universität Braunschweig, Braunschweig, Germany*

Correspondence*:
Esteban A. Hernandez-Vargas
Systems Medicine of Infectious Diseases, Helmholtz Centre for Infection Research, Inhoffenstraße 7, 38124 Braunschweig, Germany,
Esteban.Vargas@helmholtz-hzi.de

Michael Meyer-Hermann
Department of Systems Immunology and Braunschweig Integrated Centre of Systems Biology, Helmholtz Centre for Infection Research, Inhoffenstr.7, 38124 Braunschweig, Germany, mmh@theoretical-biology.de

1 SUPPLEMENTARY TABLES AND FIGURES

Supplementary Table 1. Parameter estimates using different bootstrap procedures

		Bootstrap estimates		
Bootstrap methods	Parameters (units)	2.5% quantile	Median	97.5% quantile
Parametric (lognormal assumption)	$\beta(\text{day}^{-1}\text{ffu/ml}^{-1})[10^{-7}]$	1.27	1.95	8.86
	$p(\text{ffu/ml day}^{-1}\text{cell}^{-1})$	22.76	401.48	1102.11
	$c(\text{day}^{-1})$	1.01×10^{-8}	8.40	20
	t_{inf} (hours)	3.67	5.53	12.38
Weighted bootstrap [Ma and Kosorok, 2005]	$\beta(\text{day}^{-1}\text{ffu/ml}^{-1})[10^{-7}]$	1.78	4.06	261.95
	$p(\text{ffu/ml day}^{-1}\text{cell}^{-1})$	31.80	62.91	580.69
	$c(\text{day}^{-1})$	0.18	1.05	18.76
	t_{inf} (hours)	1.68	9.49	10.79
Weighted bootstrap (fixed $c = 4.2$)	$\beta(\text{day}^{-1}\text{ffu/ml}^{-1})[10^{-7}]$	2.32	2.33	2.33
	$p(\text{ffu/ml day}^{-1}\text{cell}^{-1})$	200.33	200.34	200.47
	t_{inf} (hours)	7.025	7.026	7.03
Nonparametric	$\beta(\text{day}^{-1}\text{ffu/ml}^{-1})[10^{-7}]$	1.88	3.42	8.47
	$p(\text{ffu/ml day}^{-1}\text{cell}^{-1})$	28	107	457
	$c(\text{day}^{-1})$	0.11	1.99	9.41
	t_{inf} (hours)	5.04	7.92	10.32

1.1 PARAMETRIC BOOTSTRAP - LOGNORMAL ASSUMPTION

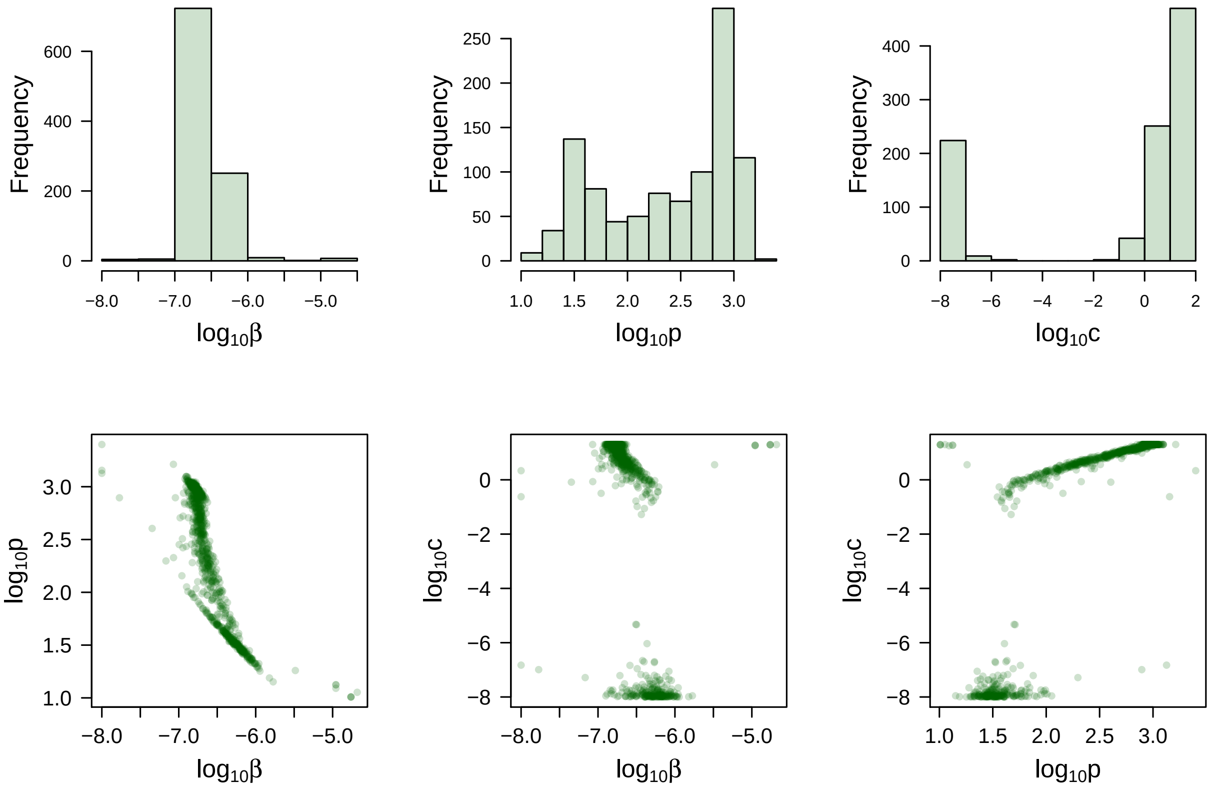

Supplementary Figure 1. Parametric bootstrap results. *Top row*: Distributions from 1000 samples estimates are presented for the three parameters: β, p and c. *Bottom row*: Scatter plot between bootstrap parameters. The parameter ρ is fixed during the bootstrapping at 0.001 [Moehler et al., 2005]. Numerical values are presented in the Supplementary Table 1.

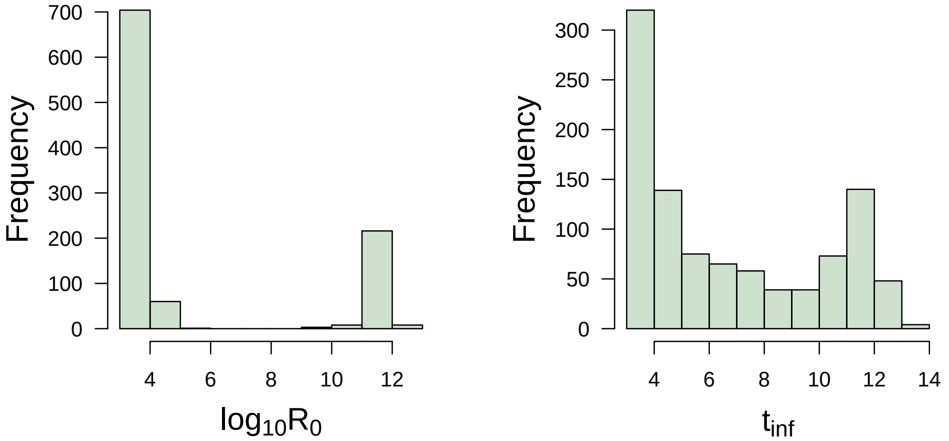

Supplementary Figure 2. Transmission measures. Bootstrap estimates of (a) reproductive number and (b) *infecting time* in hours. Numerical values can be found in the Supplementary Table 1.

1.2 NON-PARAMETRIC BOOTSTRAP

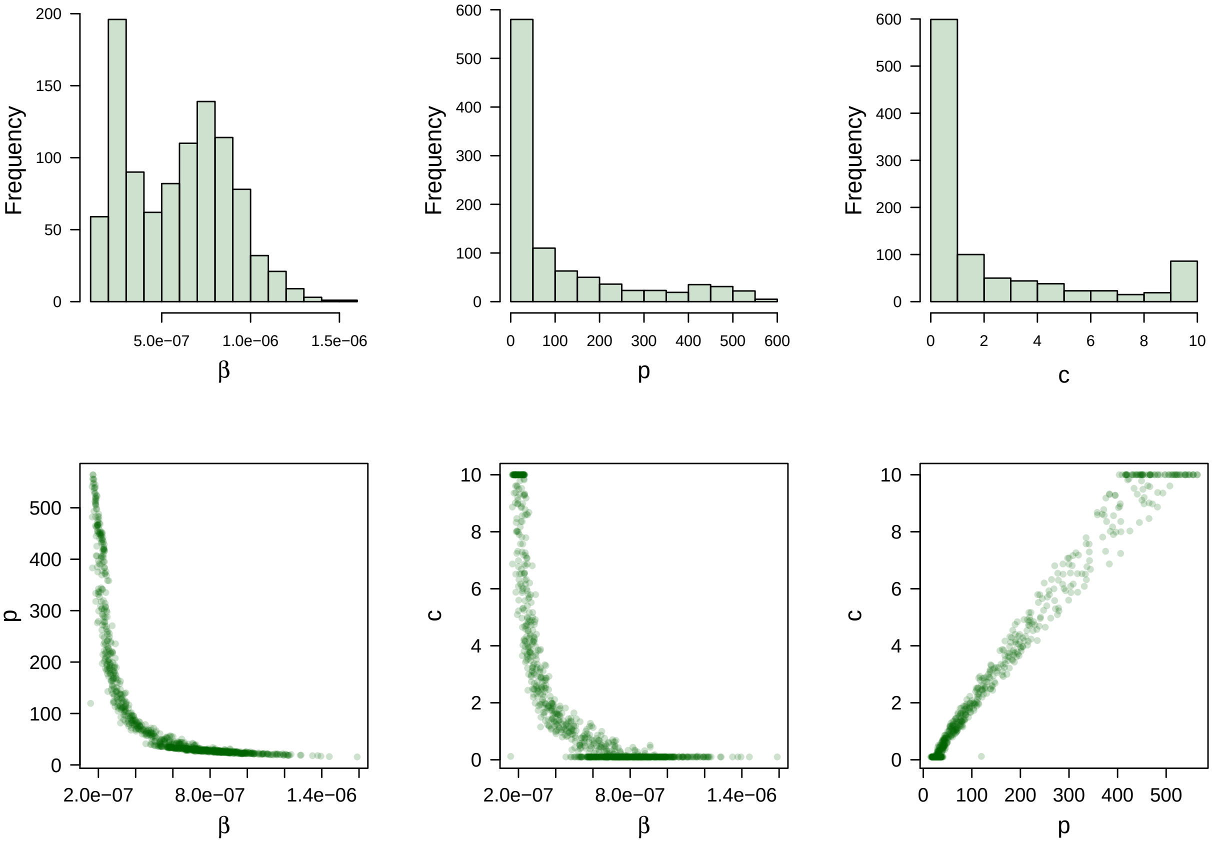

Supplementary Figure 3. Non-parametric bootstrap results. *Top row*: Distributions from 1000 samples estimates are presented for the three parameters: β, p and c. *Bottom row*: Scatter plot between bootstrap parameters. The parameter ρ is fixed during the bootstrapping at 0.001 [Moehler et al., 2005]. Numerical values are presented in the Supplementary Table 1.

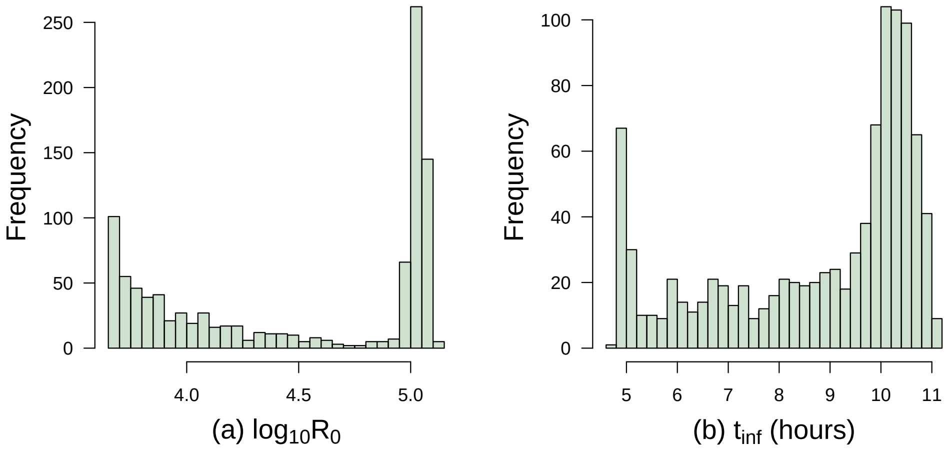

Supplementary Figure 4. Transmission measures. Bootstrap estimates of (a) reproductive number and (b) *infecting time* in hours. Numerical values can be found in the Supplementary Table 1.

1.3 WEIGHTED BOOTSTRAP - FIXING PARAMETER C

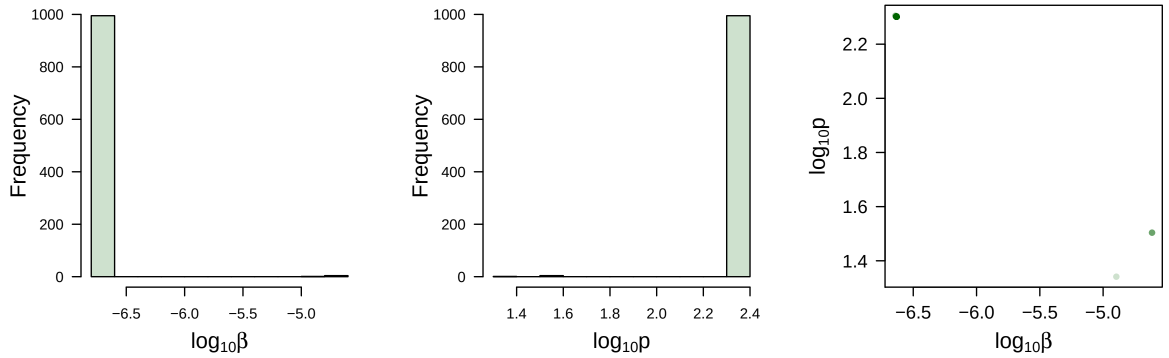

Supplementary Figure 5. Weighted bootstrap results. Distributions from 1000 sample estimates are presented for the two parameters: β, p and the scatter plot between those. Numerical values are presented in the Supplementary Table 1. None of the parameters reach the optimization boundary limits.

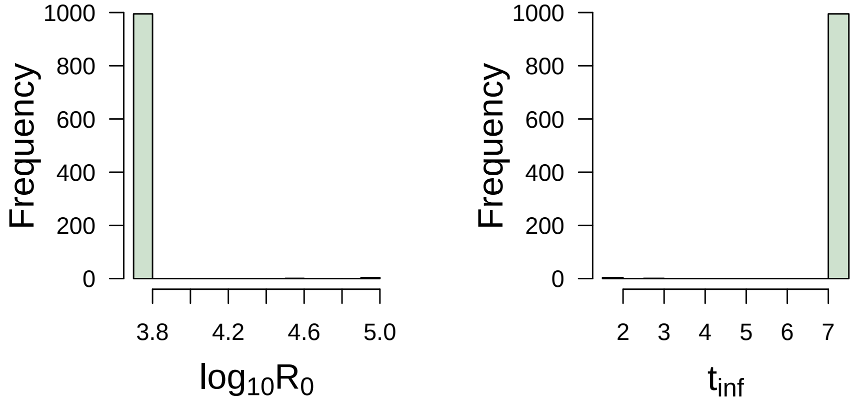

Supplementary Figure 6. Transmission measures. Bootstrap estimates of (a) reproductive number and (b) *infecting time* in hours. Numerical values can be found in the Supplementary Table 1.

REFERENCES

Ma S, Kosorok MR. Robust semiparametric m-estimation and the weighted bootstrap. *Journal of Multivariate Analysis* **96** (2005) 190 – 217. doi:http://dx.doi.org/10.1016/j.jmva.2004.09.008.

Moehler L, Flockerzi D, Sann H, Reichl U. Mathematical model of influenza a virus production in large-scale microcarrier culture. *Biotechnology and Bioengineering* **90** (2005) 46–58. doi:10.1002/bit.20363.
